# Flow Cytometry and Polymerase Chain Reaction-Based Analyses of Minimal Residual Disease in Chronic Lymphocytic Leukemia

**DOI:** 10.1155/2010/272517

**Published:** 2010-09-20

**Authors:** Sabrina Uhrmacher, Felix Erdfelder, Karl-Anton Kreuzer

**Affiliations:** Department I of Internal Medicine, University at Cologne, Kerpener Straße 62, 50937 Cologne, Germany

## Abstract

New therapeutic strategies developed recently for chronic lymphocytic leukemia (CLL) have led to remarkable treatment response rates and complete hematological remissions. This means highly sensitive and specific techniques are increasingly needed to evaluate minimal residual disease (MRD) in CLL patients. Quantitative MRD levels can be used as prognostic markers, where total MRD eradication is associated with prolonged survival. Nowadays, PCR and flow cytometry techniques used to detect MRD in CLL patients can generate reliable and quantitative results with the highest sensitivity. MRD Flow is based on four-color flow cytometry using specific antibody combinations. For allele specific oligonucleotide real-time quantification (ASO RQ) PCR individual primers are designed to detect a specific immunoglobulin heavy chain (IgH) rearrangement in each patient clone. Five comprehensive studies investigated and compared the sensitivity and specificity of both methods. Groups of patients receiving different therapies were analyzed at different time points to generate quantitative MRD levels and MRD kinetics. All studies confirmed that both methods generate equivalent results with regard to sensitivity and MRD quantification, although each method has advantages and disadvantages in the daily routine of a standard hematological laboratory. Here, we review these investigations and compare their results in the light of modern therapies.

## 1. Background

Minimal residual disease (MRD) is defined by persistence of very low levels of residual malignant cells in posttreatment cancer patients. These cells can only be detected by the most sensitive techniques available [1]. 

Formerly, reduction in tumor burden and clinical symptoms were the treatment goals in chronic lymphocytic leukemia (CLL) [2]. Although conventional therapies result in overall response rates of 40 to 60% [3, 4], only 4% of patients achieve complete remission (CR) [5]. Even the use of purine analogues such as fludarabine leads to CR, nodular CR, or partial remission in only less than half of the patients [5, 6]. The frequent persistence of a significant tumor burden after therapy is the reason why detecting MRD in CLL was previously not considered clinically important in most cases [1, 7]. 

Recently, however, the application of new therapies such as allogeneic or autologous stem cell transplantation (SCT), or monoclonal antibodies, has resulted in a significant proportion of patients attaining more profound remissions [7]. CR as defined by the National Cancer Institute sponsored working group (NCI/IWCLL) guidelines is mainly characterized by the absence of tumor and clinical symptoms such as lymphadenopathy, hepatomegaly, or splenomegaly, in combination with the absence of morphological CLL signs and stability of other parameters in peripheral blood [8, 9]. However, patients who achieved complete remission on the basis of these criteria without any morphological evidence often still have considerable infiltration of malignant cells in the bone marrow, and this can only be detected by sensitive methods [1].

Two methods are currently available for detecting MRD in CLL: multiparameter flow cytometric (MFC) analyses and polymerase chain reaction-based techniques (PCR). These methods are able to detect one malignant cell in 10,000–100,000 normal leukocytes [9, 10]. MRD detection has been used for years as a prognostic marker in other hematological disorders such as acute lymphoblastic leukemia and chronic myeloid leukemia [11, 12]. In contrast, the exact relevance of MRD detection in CLL remains to be elucidated. Several studies investigated the correlation between MRD levels and clinical outcome in CLL, providing evidence that eradication of residual disease levels can serve as a marker for prolonged survival and a better outcome [7, 13, 14]. 

For a long time it was not clear whether the application of highly sensitive MRD detection methods is capable of providing a more accurate prediction of a patient's outcome than the NCI criteria. Thus, Rawstron et al. [7] compared the event-free (EFS) and overall survival (OS) rates of patients with CR according to the NCI criteria after treatment with alemtuzumab and/or autologous SCT. In this study, patients were defined as MRD negative if CLL cells comprised less than 0.05% of all bone marrow leukocytes three months after therapy. The authors found that approximately a quarter of patients achieving complete remission according to the NCI criteria were detected as MRD positive by MFC assays. In parallel, patients who were classified as MRD positive by highly sensitive assays had significantly higher risks of relapse and also exhibited a shorter OS, independently of CR as defined by NCI criteria [7, 14]. High MRD levels also correlated with more rapid clinical progression and shorter survival than for patients with lower MRD levels [14]. However, it must be noted that patients achieving an MRD-negative status cannot be considered cured, since most of these patients develop progressive disease after a given period of time [7].

There is evidence that not only the individual MRD level provides prognostic information but also MRD kinetics [15]. In a previous study, 32 CLL patients were analyzed for their MRD level dynamics after stem cell transplantation. Although there were intraindividual differences, patients could be divided into five groups showing that MRD response within 12 months after transplantation is a strong indicator for durable remission and long-term MRD-negative survival [15]. In addition, quantitative MRD assessments during or after therapy are increasingly used to evaluate the response and adapt the duration of different types of therapeutic approaches [15, 16].

## 2. MRD Detection Methods and Principles

In contrast to other hematological malignancies, CLL is not linked to a specific structural chromosomal abnormality that could be used for MRD detection. However, in common with other B cell malignancies, it is possible to identify the malignant clone using IgH gene rearrangements or a specific cell surface antigen combination [1]. Therefore, cytogenetic methods cannot be used to detect MRD in CLL, but rather molecular or genetic assays such as PCR or flow cytometry.

### 2.1. Flow Cytometry Analyses

Flow cytometry is based on identifying cells by their cell surface antigen expression using fluorescence-labeled antibodies that bind to the surface antigens. Cells pass through laser beams where they refract the light. After antigen-antibody binding, the fluorochromes emit fluorescence light. The refracted light is analyzed by forward (FSC) and sideways scatter (SSC) to identify intracellular organization of granularity (SSC) and the cell's volume (FSC) [17]. Today, flow cytometers with two or more lasers are available, allowing the use of additional fluorochromes. Different antigen combinations measured by multiple fluorochromes are characteristic for each group of cells and vary during differentiation, migration, and proliferation. 

For flow cytometry analyses, it is critical to identify and distinguish CLL cells from other peripheral blood mononuclear cells (PBMCs), especially healthy B and T cells. Therefore, different clusters of differentiation (CD) patterns are analyzed by immunophenotyping [17].

**Figure 1:**
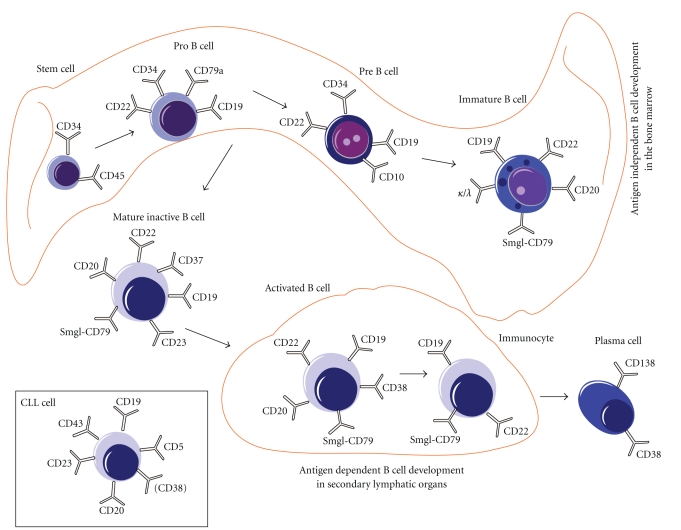


CLL cells typically express CD19, CD20, and CD23 in combination with the T cell marker CD5 in the absence of other T cells markers (Figure 1) [8]. Compared to healthy B cells, the expression of CD20 and CD79b is lower on CLL cells [18]. CD23 is an important marker for separating CLL from other non-Hodgkin lymphomas, such as the mantel cell lymphoma where CD5 and other B cell surface antigens are also expressed, but generally not CD23 [8]. The presence or absence of CD38 on the cell surface is used as a prognostic marker but varies between patients [19]. CD43 is homogeneously expressed on CLL cells, but also on mantel cell, Burkett's, and some follicular lymphomas [20]. Each CLL clone is also restricted in expression of kappa or lambda immunoglobulin light chains [21].

B cell expression patterns differ substantially during maturation. Markers such as CD10, CD20, CD21, CD23, and CD37, as well as various expression levels of Ig chains can be used to identify the different maturation stages of B cells in peripheral blood and bone marrow [17]. CD19, CD22, and CD72 are pan-B cell markers that are expressed on both immature and mature B cells. CD19 is a potent marker for B cell detection in peripheral blood because it is expressed on virtually all B cells. The presence of CD79 molecules, for example, CD79a and CD79b, is only found in B-lineage cells but varies greatly during maturation and differentiation. Cytoplasmatic CD79a is one of the earliest B cell markers. In more mature B cells both CD79 chains are expressed in combination with surface membrane bound Ig (sIg) as an sIg-CD79 complex [17].

Different antibody combinations have been tested to distinguish between CLL and healthy B cells. CD19/CD5/CD20/CD79b as well as CD81/CD22/CD19/CD5 were shown to be suitable for identifying CLL cells in peripheral blood [22, 23].

In bone marrow samples, CLL and B cells are more difficult to identify due to changes in antigen expression during differentiation. Rawstron et al. [7] achieved the best results using three different antibody combinations: CD19/CD5/CD38/CD79 plus CD19/CD5/CD20/CD79b and CD19/CD5/CD38/CD20. 

Sensitivity and specificity also depend on the minimum number of cells required to identify a cell population as being CLL cells in flow cytometric analyses. Contamination rates or the number of cells acquired in a sample can be limiting factors for detection [10]. The absolute number of cells required to characterize a cell population is defined as the absolute specificity threshold [24].

### 2.2. Polymerase Chain Reaction (PCR)

Real-time quantitative PCR (RQ-PCR) is used to generate quantitative MRD results. The principle of this PCR is an indicator that generates a detectable fluorescence signal during or after each PCR cycle, thus providing quantitative results directly after measurement [25]. Three types of fluorescence formats are available: DNA intercalating fluorochromes, hydrolysis, or hybridization probe techniques [25]. The principle for all of these techniques is the increase in fluorescence during the exponential extension/elongation of the DNA product. 

Intercalating dyes are mainly used for nonspecific detection. The dye binds to double-stranded DNA, which leads to an increase in fluorescence during the elongation period. Since new DNA strands are generated exponentially, the dye can bind more substrate [25]. Hydrolysis and hybridization probe techniques can be used for specific PCR product detection.

Hydrolysis probes use the activity of the *Thermus aquaticus (Taq)* polymerase to detect and quantify a specific PCR product. When the hydrolysis probe is conjugated with a reporter and a quencher fluorochrome is located within the target sequence, as long as the DNA strand is intact, the quencher fluorochrome absorbs the reporter fluorochrome due to their close contact. During the elongation step, the hydrolysis probe is replaced by Taq-polymerase and hydrolyzed. As a consequence, both fluorochromes loose their proximity, the reporter fluorochrome is no longer quenched and becomes detectable [25]. Again the detectable fluorochrome increases exponentially in every PCR cycle. Hybridization probes work with a donor and acceptor fluorochrome. During the annealing phase and the early elongation step, both fluorochromes are in contact and one can detect emission from the donor fluorochrome [25].

To detect MRD, it is necessary to identify DNA sequences that are unique to CLL cells. Such a PCR target can be the immunoglobulin gene (Ig), for example, Ig heavy chain, Ig kappa, or Ig lambda. During antigen-independent maturation in the bone marrow, the antibody variability increases due to rearrangement of germline variable (V), diversity (D) and joining (J) gene segments of the Ig [26]. This allows a specific VDJ “fingerprinting” combination for each immature (pre-) lymphocyte [25]. Therefore, in principle, all malignant cells in one patient exhibit the same clonal origin with identically rearranged Ig genes. Identifying the patient-specific Ig arrangement is the basis for developing the PCR technique, which can then generate very sensitive results using specific primers for each patient clone. A problem associated with using specific immunoglobulin genes can be somatic hypermutations that change the primer binding region after the lymphocytes contact antigens. Fortunately, although somatic hypermutations are high in myeloma (median 8%), they are low in common CLL (2%) [27].

Gene rearrangement often leads to differentiation into subclones, for example, secondary Ig gene rearrangements, ongoing VH to DH-JH joining or VH replacement, which can occur during the time between diagnosis and relapse. Mostly, the D-J junctional region remains unaffected during the secondary rearrangements, and therefore primers are designed around this region [25]. However, if oligoclonal clones are found at the time of diagnosis [13], it is unclear which clone should be monitored by PCR for early identification of relapse. If using only one oligonucleotide primer, the PCR probes can loose specificity for the chosen target region and the clone causing the relapse is possibly not identified. This may lead to false negative results, which is a known problem in childhood acute lymphoblastic leukemia (ALL) [28]. Thus, if clonality is detected in a CLL patient, two Ig PCR targets can be used to minimize false negatives.

## 3. Low-Sensitivity Methods: Two-Color Flow Cytometry and Consensus PCR

### 3.1. CD5^+^/CD19^+^ Flow Cytometry

The first flow cytometric analysis for detecting MRD in CLL, CD5^+^/CD19^+^ flow cytometry, was an insensitive and only qualitative technique, since single antigens were insufficient to separate CLL cells from normal B cells [7, 29, 30]. Two-color cytometry was used to detect the coexpression of a B cell marker such as CD19 or CD20, and CD5 (which under normal circumstances is only expressed on T cells) in conjunction with monoclonality detected by surface light chain expression [1, 31]. Patients with less than 25% of CD19^+^CD5^+^ cells/total CD19^+^ cells in peripheral blood and <15% of CD19^+^CD5^+^/total CD19^+^ cells in bone marrow were defined as MRD negative [29]. Using coexpression of CD20 and CD5, the cutoff point was defined as 10% to identify residual CLL cells [31]. 

Not only is this form of assessment not quantitative, but sensitivity is also very low and variable. For example, samples detected as MRD negative in the two-color flow analysis might have 100-fold higher levels of disease than MRD samples detected as MRD positive by newer and more sensitive techniques [7]. The sensitivity of two-color flow cyometry is also limited by the presence of normal B cells in the peripheral blood or B cell progenitor cells in the bone marrow, especially when CLL cell numbers are very low [1, 7]. 

Two-color flow cytometry usually detects one CLL cell in 200 healthy B cells [32]. Some studies also tested the expression of CD79b and CD20, but interpatient variation in antigen expression means that two-color flow cytometric analyses are not informative for all patients, regardless of the chosen antibody combination [7, 33, 34]. Another limitation is the rate of false positive results after autologous CD34^+^ sorted PB stem cell transplantation. On average, 36% of normal CD19^+^-B and progenitor cells also exhibit CD5 coexpression within the first two months, persisting in some patients for over one year after transplantation [35].

### 3.2. Consensus Primer PCR

For this technique, a standard oligonucleotide primer set comprising two primers is used to amplify the third complementarily region (CDRIII) of the IgH gene. The first primer detects the consensus JH-sequence region, and the second one the family specific framework region [36]. For CLL detection, framework region, one or three are possible targets [7, 22]. Consensus PCR can be used for qualitative MRD detection giving less prognostic information [1] and achieves variable sensitivity of around 10^−3^ [7, 24], with sensitivity limited by the presence of normal leukocytes [1]. Rawstron et al. found that consensus PCR detects CLL cells when they represented at least 2% of all B cells; otherwise the primer also binds to the germline DNA of other healthy cells [7]. Other limitations are B cells with a polyclonal background [24, 36]. The detection rate of consensus PCR also depends on the framework or JH region used [37].

CLL patients can be divided into mutated or unmutated IgH subgroups [38], and since mutations can abrogate binding of the consensus primers, MRD detection with standard consensus PCR was only possible in approximately 70%–80% of all patients [7]. To increase the sensitivity and specificity of PCR detection, specific primer sets were developed by the European BIOMED-2 collaborative study group. These primers increased sensitivity of PCR detection in non-Hodgkin lymphomas to 82%–100% and potentially the majority of CLL. [39] In a comparative analysis by Böttcher et al. [24] on patients who had received allogeneic or autologous SCT, consensus PCR was compared to MRD Flow using ASO RQ-PCR as a reference method. Consensus PCR was shown to be inferior to both the other techniques with respect to quantification and sensitivity [24]. Using consensus PCR, the authors achieved sensitivity ranging from 2.2 × 10^−4^ for patients with a monoclonal CLL background and up to 5.2 × 10^−3^ for a polyclonal CLL background. Nevertheless, this was superior to the studies from Rawstron et al. [7], probably due to reductions in healthy B cells after SCT enhancing specificity. The limitation of a polyclonal B cell background was also shown in other studies [36]. Due to the drawbacks of consensus PCR for MRD detection and the development of more efficient treatments, other quantitative PCR techniques such as ASO RQ-PCR, and more sensitive flow cytometric analysis have now replaced consensus PCR in MRD diagnostics [7, 24]. 

## 4. High-Sensitivity Methods: Multiparameter Flow Cytometry (MFC) and ASO IGH PCR

### 4.1. Multiparameter Flow Cytometry

Further therapeutic developments and the idea of using MRD as a prognostic marker made it necessary to find methods that are not only more sensitive but also quantitative [22]. One of the major problems of the commonly used CD19+/CD5+ flow cytometry analyses was that one could only analyze the expression of two antigens. This proved not enough to reliably identify CLL cells nor achieve good separation of healthy B cells and CLL cells due to their overlapping antigen pattern.

The advantages of multicolor flow cytometry are that it can differentiate between cell groups by using a greater number of antibodies, measure large numbers of cells, and identify very small cell populations, all important to reach higher specificity. It can also be used for peripheral blood and bone marrow analyses [7].

In 2001, Rawstron et al. [7] analyzed different flow cytometry approaches based on investigating the variable antigen with advanced techniques. Using blood from patients posttreatment with alemtuzumab and/or autologous stem cell transplantation, the authors compared conventional four-color flow cytometry, which detected the cell surface combination CD19/CD5/*λ*/*κ*, with MRD Flow employing a specially developed antibody combination and gating strategy. Kolmogorov-Smirnov calculations were used to identify the extent of separation of each antibody combination tested [7]. The MRD Flow antibody combination achieving the highest sensitivity of all tested antibody combinations was CD19/CD5/CD20/CD79b for peripheral blood.

To define the specificity and sensitivity of the MRD Flow, the authors designed dilution series where CLL cells were mixed with healthy leukocytes in concentrations from 1 : 1 to 1 : 16,384 [7]. These dilutions were also used for conventional four-color flow cytometry measurements and consensus PCR performed with a JH and FR3 consensus primer. For flow cytometry experiments, a minimum of 0.5 × 10^6^ leukocytes were incubated with different antibody combinations, and each test was analyzed between 50000 and 800000 cells using a Becton Dickinson FACSort instrument.

Comparing the sensitivity of both flow cytometry methods in the dilution studies revealed that the MRD Flow was 2 logs more sensitive than the conventional four-color analyses or consensus PCR. The median was 0.005 for MRD Flow, 0.4 for four-color flow cytometry, and 0.6 for PCR [7]. The sensitivity of the two-color flow cytometry was assessed as 1.2. In addition, the specificity of the MRD Flow for identifying CLL cells was higher than for conventional four-color flow cytometry. Whereas four-color flow cytometry could only detect an abnormal kappa-lambda ratio in CD5^+^ B cells when about 30% were CLL cells, the MRD Flow was able to detect CLL cells when they only represented around 1% of all B cells.

This study used a specific gating strategy to completely separate CLL from normal B cells or B cell progenitors. MRD Flow was also used to monitor patients with CR according to NCI criteria. The authors showed that the absence of clinical symptoms was not identical to the absence of CLL cells in peripheral blood or bone marrow. Thus, MRD Flow analyses provided information about incomplete eradication or relapse of CLL, allowing prognosis of event-free survival or disease progression. 

A study by Böttcher et al. [22] investigated the possibility of using MRD Flow and ASO RQ-PCR to quantify MRD levels in CLL patients after treatment with immunochemotherapy, including the CD20 monoclonal antibody rituximab. Since rituximab has proven to be very effective in CLL treatment and therefore has become a component of current standard therapy, it was essential to test whether all methods used for MRD detection still work at the presence of this antibody [40, 41].

For their study, the authors analyzed 530 samples from 63 patients enrolled in the CLL8 trial of the GCLLSG. At different time points, samples were tested for MRD levels (during therapy, one and three months after therapy, and in three-month intervals thereafter). All patients included in this study were randomized and divided in two groups, each receiving different therapies. In the first group, patients underwent six courses of a chemotherapy regime combining fludarabine, cyclophosphamide, and rituximab (FCR), whereas patients in the other group were treated with fludarabine and cyclophosphamide (FC) alone [22]. Samples were classified as MRD negative if MRD levels were 10^−4^ or less, and as MRD positive when CLL cell numbers were greater than 10^−4^ [22]. Detection of MRD levels at the threshold of 10^−4^ is the given standard for MRD detection according to the IWCLL guidelines [8].

The authors showed that MRD Flow and ASO RQ-PCR gave concurring qualitative results in 85.3% (452 samples) of all measured samples. This included 278 samples that were MRD positive for both methods within the quantitative range; 34 samples that were simultaneously positive outside the quantitative range (positive results that cannot be precisely quantified, for example, positive PCR samples that started with a very low template number due to marginal CLL cells), and 140 samples that were MRD negative. ASO RQ-PCR proved to be more sensitive for qualitative MRD detection, identifying 72.8% (386) as MRD positive whereas MRD Flow only detected 59.6% (316) as MRD positive. All 316 MRD-positive samples tested by MRD Flow (within and outside the quantitative range) achieved a median MRD level of 3.3 × 10^−3^. The median quantitative range for the 386 MRD-positive ASO RQ-PCR samples was 2.2 × 10^−4^. 

In the 278 concurring ASO RQ-PCR/MRD Flow samples within the quantitative range, MRD Flow reached a median quantitative range of 8.2 × 10^−3^ (range: 6.1 × 10^−5^ to 9.5 × 10^−1^), which correlated very well with the achieved ASO RQ-PCR median range of 8.5 × 10^−3^ (range: 5.0 × 10^−5^ to 6.5 × 10^0^). 

Discordant results were achieved in 14.7% (78) of samples. Most of these samples (67) were classified as outside the quantitative range, and ASO RQ-PCR generated more positive results than MRD Flow. Quantifiable MRD levels were detected in seven samples by ASO RQ-PCR but not by MRD Flow. In these cases, insufficient leucocytes were available to allow detection of CLL cells by MRD Flow. Thus, the MRD levels matched or were lower than the MRD Flow sensitivity.

In summary, MRD Flow generated equivalent quantitative results compared to ASO RQ-PCR according to the standard given by the NCI guidelines but had a lower sensitivity for qualitative detection (78% of all samples were ASO RQ-PCR positive but only 59.6% were MRD Flow positive). Such information about the sensitivity and quantification ranges of both methods is important for interpreting MRD data and avoiding unquantifiable results.

To find out whether rituximab influences MRD detection sensitivity, Böttcher et al. correlated the results of MRD Flow for both therapeutic arms. Different antibody combinations were tested, and ASO RQ-PCR was used as a reference method since it is not affected by treatment with antibodies [22]. This comparison showed that (i) patients with the FCR combination had significant lower MRD levels detected by MRD Flow and (ii) rituximab had no effect on the specificity, sensitivity, or quantification of MRD Flow compared to ASO RQ-PCR [8]. Due to these findings, it was necessary to analyze whether the antibody combinations without CD20 produced superior results; but in fact antibody combinations including CD20 were as effective as combinations without CD20. To explain their results, the authors analyzed the kinetics of CD20 expression in CLL cells as well as benign B cells during and after treatment with rituximab. They concluded that during therapy the disappearance of CD20 on CLL cells coincides with a depletion of benign CD20^+^ B cells, thus reducing the B cell background and influencing the specificity of MRD Flow. Sayala et al. were more critical on this point, advising against using CD20 combinations due to rituximab's effect on normal B cells [1].

The coexistence of many flow cytometry assays such as MRD Flow, conventional four-color, and CD19^+^/CD5^+^ flow cytometry makes it difficult to compare factors such as therapy response. To address this problem, Rawstron et al. [10] developed an international standard for using flow cytometry in MRD diagnostics, to facilitate comparison between different flow cytometry techniques as well as PCR assays. The authors used MRD Flow as well as ASO RQ-PCR and also examined which kind of patient material (peripheral blood or bone marrow) is required for measurements during different times of treatment. Eradication of malignant CLL cells from peripheral blood during therapy with monoclonal antibodies is faster than significant clearance in the bone marrow. This information is important for making decisions about the time point and material used for MRD assessments during monitoring [1]. For example, intratherapeutic monitoring optimally requires bone marrow analyses. 

These standards were based on examining 728 samples of peripheral blood and bone marrow using a cutoff point of 0.01%. Similarly to other studies, the authors found well-correlated results for MRD Flow and ASO RQ-PCR in 94.7% of cases [7, 22]. The accuracy for MRD Flow was 95.0%, and again ASO RQ-PCR showed higher sensitivity for MRD detection [10].

Several antibody combinations were tested for detecting CLL cells in peripheral blood, identifying the combinations of CD20/CD79b/CD19/CD5, CD20/CD38/CD19/CD5 and CD22/CD81/CD19/CD5, as those with the highest sensitivity and significant correlation between MRD Flow and ASO RQ-PCR. The correlation between materials depended on the treatment type and time of assessment [10]. Discordant results were found in samples from patients during or shortly after treatment with alemtuzumab whereas material from patients not receiving alemtuzumab gave concordant results in 91.6% of samples. In some cases, peripheral blood was shown to be equivalent or more sensitive than bone marrow. This likely occurs mainly in the regeneration phase when increasing proliferation of normal progenitor cells in the bone marrow leads to a higher background of cells. Nevertheless, bone marrow analyses were necessary for MRD detection during and within the first three months after therapy with alemtuzumab or rituximab [23], since neoplastic B cells as well as normal cells can be depleted in peripheral blood for several months after treatment but can still persist in the bone marrow [10].

In summary, MRD Flow is capable of detecting one CLL cell in 10^4^ healthy leukocytes with a sensitivity as effective as ASO RQ-PCR within the given standard according to the international consensus and IWCLL guidelines [8, 22]. This means that MRD Flow reaches the diagnostic standard. Given antibody combinations, as well as a special gating strategy providing detailed instructions for identifying CLL cells [10], are necessary to separate CLL cells from healthy cells and enhance sensitivity and specificity. 

However, the technical background is important since flow cytometry analyses are influenced by several factors that decrease sensitivity and specificity [24, 42]. Such factors include unspecific antibody binding, which leads to false positive results or contamination of the flow system, which leads to suboptimal detection of CLL cells. Even different procedures for erythrocyte lysis can influence flow cytometric analyses [24, 43, 44].

Compared to other methods, MRD Flow assessments generate results very rapidly (Table 1). Additionally, MRD Flow is not as expensive as the sensitive PCR methods and is available in most hematological laboratories. Using this sensitive flow cytometry technique, it is also not necessary to know the exact immunophenotype of the CLL patient clone. The problem of variable antigen expression patterns between different patients can be reduced by using standardized antibody combinations that are highly sensitive for most of the variations although patients with atypical antigen profiles remain a problem for flow cytometry detection [7].

### 4.2. ASO IGH RQ-PCR

The allele specific oligonucleotide immunoglobulin heavy chain real-time quantitative polymerase chain reaction (ASO IGH RQ-PCR) is an MRD detection method based on designing individual oligonucleotide primers for each patient clone. This generates high specificity and sensitivity of up to 10^−5^ [1, 24, 45]. However, this high sensitivity depends on sequencing samples for each patient before designing the necessary fluorogenic probe. Three different options for designing an ASO RQ-PCR are described [25]. 

The first approach is the ASO probe, where the individually designed probe is positioned at the tumor-specific gene sequence in the CDRIII region of the IgH gene rearrangement [22]. The forward and reverse primers are conventional and positioned in the germline sequences flanking the tumor specific sequence. Primer competition between the MRD target and comparable Ig rearrangements in normal cells might lead to decreased fluorescence intensity if the number of CLL cells in the sample is very low.

The two other approaches are based on generating an ASO forward or ASO reverse primer. The ASO forward primer is positioned within the tumor-specific sequence in combination with a conventional germline probe and reverse primer. The probe is normally positioned in the J gene segment. The increase in fluorescence correlates with the percentage of CLL cells in the sample due to the exponential sequence-specific DNA amplification [25].

The probe used for the ASO reverse primer approach is normally designed for the V gene segment. It works like the ASO forward primer but in the opposite orientation. Here, the reverse primer is positioned at the tumor-specific sequence.

The ASO forward primer has advantages over the ASO reverse primer approach [25]; first is the location of the probe. The ASO forward primer locates to the J segment. Due to the lower number of J gene segments than V gene segments, where the ASO reverse primer binds, a lower number of probes have to be designed for the ASO forward primer approach than for ASO reverse primers [25]. Furthermore, ASO reverse primers are more prone to target loss due to V_H_-replacement and somatic hypermutations, which are often found in V gene segments [46, 47], although somatic hypermutations are rare in CLL patients [27]. An advantage of the ASO reverse primer approach over the ASO forward primer might be increased sensitivity due to the higher number of V gene segments. However, no data supporting this hypothesis have as yet been published [25]. 

Many studies report that ASO RQ-PCR is the method with the highest sensitivity for MRD detection in CLL [7, 10, 24]. Böttcher et al. [24] compared the efficiency of consensus PCR, ASO RQ-PCR, and MRD Flow for detecting MRD in CLL cells after allogeneic and autologous SCT. The ASO primers were designed for the hypervariable N-D-N region of the IgH-CDR3, the antibody combination for the MRD Flow assay comprised CD19/CD5/CD20/CD43, and consensus PCR used an FR1-IgH primer set. From the 74 patients included in this study, the authors analyzed 92 samples from 40 patients in parallel using ASO RQ-PCR and MRD Flow. ASO RQ-PCR detected MRD in 61 samples whereas MRD Flow only detected MRD in 47 samples. All MRD Flow^+^ samples were also ASO RQ PCR^+^. Concordant positive results were generated in 51.1% (47 samples) of all samples whereas 33.7% (31 samples) gave concordant negative results [24]. Thus, the same qualitative results (MRD^−^ or MRD^+^) were reached with 84.8% (78 samples) of all examined samples. The quantitative MRD levels detected in the 51.1% of concurring positive samples were well correlated (*r* = 0.93, *P* < .0001) [24] with a median level of 3.2 × 10^−3^ for MRD Flow and 6.1 × 10^−3^ for ASO RQ-PCR [24]. 

Eight patients (14 samples) were positive for ASO RQ-PCR but negative for MRD Flow. These patients had very low levels of disease with a median range of  5.2 × 10^−5^ (range 1.3 × 10^−5^ to 1.6 × 10^−3^). In two of these patients, both methods detected MRD at different time points in the study [24].

Taken together, they found well-correlated results between both methods within the guidelines set for sensitivity, which was attained by both techniques. ASO RQ-PCR could detect lower MRD levels than MRD Flow, and therefore ASO RQ-PCR is the method of choice for identifying very low levels of disease. The role of consensus PCR was again shown to be inferior [24]. 

In the above described study [22], when comparing MRD Flow and ASO RQ-PCR in rituximab-treated patients, the ASO RQ-PCR was designed with the specific ASO primer matching the hypervariable N-D-N region of the individual CLL clone, plus conventional reverse and forward primers. Amplification of polyclonal DNA from pooled healthy donor samples in every PCR cycle was used to calibrate specificity. Optimal sensitivity and specificity were reached by analyzing different temperatures for the annealing step [22]. Again the results revealed concordant qualitative and quantitative MRD detection for both methods, and again ASO RQ-PCR proved to be more sensitive for lower MRD levels up to 10^−5^ [10, 24]. The authors attested good specificity and quantification for both methods in clinical diagnostics, as long as the recorded results were within the sensitivity and quantitative ranges, as calculated using serial dilutions.

Rawstron et al. [10] also identified ASO RQ-PCR as the method with the highest sensitivity. They compared MRD Flow and ASO RQ-PCR in 57 samples from 39 patients. MRD Flow was performed as described above, while ASO primers were designed for the CDR3 region and the DNA was isolated and amplified according to the BIOMED-2 protocol [36]. Serial dilutions were used to create a standard, and sensitivity and specificity were defined as the last dilution where target DNA was detectable [10]. The authors showed that both methods reached equal efficacy for MRD detection at a cutoff of 0.01%, but ASO RQ-PCR had greater sensitivity with a limit of MRD detection below 0.001%.

An advantage of ASO RQ-PCR compared to conventional consensus PCR is that specifically designed oligonucleotides can overcome interference by healthy B cells that have similar IgH segments [7], explaining the higher sensitivity of ASO RQ-PCR. However, MRD detection with ASO RQ-PCR is expensive and time consuming, thus not available in every diagnostic laboratory.

## 5. Discussion

If the more potent therapies developed recently to treat CLL are to achieve their full potential, highly sensitive MRD detection is essential to obtain prognostic information, evaluate therapeutic responses, and monitor posttreatment patients [7, 10, 13, 14]. Rather insensitive and only qualitative methods such as CD19^+^/CD5^+^ flow cytometry or consensus PCR are no longer sufficient for accurately assessing MRD in CLL [10]. Instead, multiparamer flow cytometry (MRD Flow) and allele specific oligonucleotide immunoglobulin heavy chain real-time quantitative polymerase chain reaction (ASO IGH RQ-PCR) techniques have been validated as being not only more sensitive, but also quantitative by five extensive studies reviewed here [7, 10, 15, 22, 24].

To ensure optimal application of these techniques, a number of working parameters are needed, such as defining which patient material to use; establishing sensitivity, specificity, quantification ranges, and correlation with patient prognosis; setting international standards for comparing results in different settings and between methods; defining ease of application, costs, required expertise, and whether the cytometry assays are functionality independent of previous therapies, including monoclonal antibodies. 

All the studies directly compared MRD Flow and ASO RQ-PCR with conventional flow cytometry and PCR, analyzing peripheral blood and/or bone marrow from patients following different standard treatments, including rituximab [22], alemtuzumab [7], or allogeneic/autologous SCT [7, 15, 24]. Previous investigators had already confirmed that using the appropriate patient material is critical, since specificity may decrease due to the treatment, the sampling time point, or technical limitations [10]. For prognostic information, not only the sensitivity of the chosen MRD detection technique is important but also MRD kinetics and the patient sample type [15], since CLL disease kinetics are different for peripheral blood and bone marrow [1]. MRD assessment during, or shortly after, administrating monoclonal antibody regimes optimally requires bone marrow analyses [10, 23]. However, evaluation of therapeutic response and prognostic values can also use peripheral blood without losing specificity or sensitivity, a significant benefit for patients since blood withdrawal is not as invasive as bone marrow aspiration. 

It is essential to standardize MRD assays to compare therapeutic treatments and use MRD as a prognostic marker. In 2007, Rawstron et al. outlined an international standard approach to flow cytometry analyses [10], that is, in line with the former international guidelines [8]. This included proposals for blood processing, antibody combinations, and gating strategies [10]. Flow cytometry analyses that can use whole blood and different protocols for blood preparation have been applied although the international standard recommends preparation with whole blood lysates to eliminate erythrocytes. Density-gradient centrifugation should not be used for cell preparation, since quantitative MDR analyses were inaccurate due to variable cell recovery, especially in bone marrow [10].

For standardized MRD detection, settings were chosen that are not, or only marginally, influenced by the presence or absence of any therapeutic components, including monoclonal antibodies. Of particular significance was the observation that MRD Flow analyses are unaffected by the presence of monoclonal antibody rituximab [22]. 

Together with gating strategies, specific antibody combinations were used in MRD Flow to separate CLL cells from other PBMC [7, 22]. Previously, high numbers of healthy cells limited CLL cell detection [44]. With the more advanced multicolor flow cytometry, it is now possible to measure large numbers of cells, overcoming the problem of interference by too many healthy cells, and thus increasing specificity.

Several studies reported that both MRD Flow and ASO RQ-PCR are able to detect MRD levels within the international guideline threshold, with high accuracy. When MRD levels are between 10^−4^ and 10^−5^ or even lower, only ASO RQ-PCR can provide qualitative detection, since it proved to be more sensitive than MRD Flow. However, both methods gave correlating results, therefore highlighting the clinical relevance of both MRD Flow and ASO RQ-PCR.

Higher sensitivity of ASO RQ-PCR is due to the individual primer designed for every patient clone. Interference by other healthy B cells is therefore very low, allowing high sensitivity even for very low disease levels. The specificity depends on the length of the gene segment and the annealing temperature [22, 25]. Analyzing complete V-D-J rearrangements in the IgH gene showed higher sensitivity than analyzing the smaller V-J rearrangement segment [25, 48]. However, since specific primers address only one rearranged IgH gene sequence, the risk of target gene loss due to ongoing rearrangements in the IgH region is high and leads to reduced specificity. In addition, normal B cells with similar VDJ rearrangements can produce false positive results [25]. 

Certainly both techniques have advantages and disadvantages. Although MRD Flow is available in most laboratories and is less expensive than ASO RQ-PCR, its sensitivity is somewhat lower than ASO RQ-PCR, and training and expertise are important to achieve reliable results. The sensitivity and specificity of MRD Flow can be reduced by some parameters that generate false negative or false positive results. Using whole blood for MRD Flow analyses, blood processing or the use of different antibodies are highly relevant for the quality of the results. In general, monoclonal antibodies are used for MRD detection since it is technically easier to develop standardized batches of monoclonal rather than polyclonal antibodies. A major disadvantage of ASO RQ-PCR the high costs for IgH sequencing although blood from patients in clinical trials is routinely sequenced to evaluate IgH mutations or find prognostic markers [10]. Also, due to differences in the quality of DNA samples, it is necessary to normalize their amount and quality, which can be performed by using albumin genes, for example, as internal references [24].

In summary, MRD can be effectively determined in CLL patients by multiparameter flow cytometry, since within a sensitivity range of down to 10^−4^, it provides information as accurate as ASO RQ-PCR. However, ASO RQ-PCR offers even higher qualitative sensitivity, which might be relevant if the therapeutic aim is complete eradication of the disease. Since multiparameter flow cytometry is widely available and no standard treatment can cure CLL, this technique should be considered a routine method. In contrast, ASO RQ-PCR analyses should be limited to a few specialized laboratories that can cope with the necessary expenditure; presently it is only indicated within clinical trials. Possibly, new antibody combinations and, most importantly, very recent technological improvements will further enhance the sensitivity of flow cytometric analyses [24].

## Figures and Tables

**Table 1:** Overview about the most important differences between different MRD detection methods.

	MRD Flow	ASO RQ-PCR
sensitivity	10^−4^	10^−4^–10^−5^

specificity	depends on	depends on
	(i) measured cell type	(i) annealing temperature
	(ii) B cell background	(ii) length of amplified DNA
	(iii) antibody quality	

method	(i) surface antigen detection	(i) detection of amplified DNA with individual primer
	(ii) possible antibody combinations:	(ii) possible targets:
	(1) CD20/CD79b/CD19/CD5	(1) Ig heavy chain
	(2) CD20/CD38/CD19/CD5	(2) Ig kappa
	(3) CD22/CD81/CD19/CD5	(3) Ig lamda

limiting factors	(i) high normal B cell background	(i) somatic hypermutation (rare in CLL)
	(ii) antibody quality	(ii) loss of target gene

advantages	(i) high number of cells can be measured	(i) highest sensitivity of all available methods
	(ii) cost effective	(ii) low interference with normal B cells
	(iii) available in day-to-day business	
	(iv) rapid (approx. 1 hour)	

disadvantages	(i) knowlegment for reliable results	(i) expensive (sequencing, PCR primers)
	(ii) contamination of the system	(ii) time-consuming
		(iii) not available in every diagnostic laboratory
